# Transposable Element Expression Profiles in Premalignant Pigment Cell Lesions and Melanoma of Xiphophorus

**DOI:** 10.3390/genes15050620

**Published:** 2024-05-14

**Authors:** Luca Münch, Frederik Helmprobst, Jean-Nicolas Volff, Domitille Chalopin, Manfred Schartl, Susanne Kneitz

**Affiliations:** 1 Neurology Asklepios Klinik Barmbek, Rübenkamp 220, 22307 Hamburg, Germany; luca.muench@yahoo.de; 2 Institute of Neuropathology, Philipps-University Marburg, 35037 Marburg, Germany; helmprob@staff.uni-marburg.de; 3 Institut de Génomique Fonctionnelle de Lyon (IGFL), 69007 Lyon, France; jean-nicolas.volff@ens-lyon.fr; 4 University of Bordeaux, CNRS, IBGC, UMR 5095, 33000 Bordeaux, France; domitille.chalopin@gmail.com; 5 The Xiphophorus Genetic Stock Center, Department of Chemistry and Biochemistry, Texas State University, San Marcos, TX 786666, USA; 6 Developmental Biochemistry, University of Würzburg, 97974 Würzburg, Germany; 7 Biochemistry and Cell Biology, Biocenter, University of Würzburg, Am Hubland, 97074 Würzburg, Germany; susanne.kneitz@uni-wuerzburg.de

**Keywords:** transposable elements, melanoma, fish model

## Abstract

Transposable elements (TEs) are characterized by their ability to change their genomic position. Through insertion or recombination leading to deletions and other chromosomal aberrations, they can cause genetic instability. The extent to which they thereby exert regulatory influence on cellular functions is unclear. To better characterize TEs in processes such as carcinogenesis, we used the well-established Xiphophorus melanoma model. By transcriptome sequencing, we show that an increasing total number in transposons correlates with progression of malignancy in melanoma samples from Xiphophorus interspecific hybrids. Further, by comparing the presence of TEs in the parental genomes of *Xiphophorus maculatus* and *Xiphophorus hellerii*, we could show that even in closely related species, genomic location and spectrum of TEs are considerably different.

## 1. Introduction

Malignant melanoma accounts for 60% of skin cancer deaths, making it the deadliest of all known types of skin cancers [1]. Over the past several decades, both incidence and prevalence have increased at an alarming rate across genders, ages, and countries [2,3]. One characteristic of most cancers is genomic instability. While many intrinsic as well as extrinsic sources of genomic instability are known, the roles of transposable elements (TEs) have been the subject of scientific discourse for years [4,5].

TEs were first described by Barbara McClintock. McClintock‘s and Britten and Davidson‘s research results pointed to a wide regulatory function of TEs without neglecting the potentially deleterious effect of their transpositions [6,7,8]. The identification of regulatory elements within transposable sequences changed the evaluation of their genomic function [9,10] and raised questions about their role in processes such as carcinogenesis [11]. The mutagenic potential of TEs is undisputed and well documented in many animal models [12,13,14,15].

In this study, we used the Xiphophorus melanoma model, which is characterized by the reproducible generation of pathologically uniform melanocytic lesions that develop after simple crossing experiments [16]. Belonging to the group of evolutionary mutant models, Xiphophorus has naturally evolved disease genes. It is an established system to study the molecular processes underlying the neoplastic transformation of melanocytes and the progression of the disease [17]. Malignant lesions show high ultrastructural, histological, and molecular similarities to human malignant melanoma [16,18,19,20]. The driver of cancer development in this model is a tumor locus (Tu locus) on the sex chromosome/linkage group 21 that many, but not all Xiphophorus species possess. It encodes a receptor tyrosine kinase called Xiphophorus-melanoma-receptor-kinase (Xmrk) that arose from a local duplication of a fish ortholog of the human epidermal growth factor receptor (EGFR) gene [21,22]. In wildtype fish, the oncogenic activity of the Tu locus is suppressed by the action of an autosomal regulator locus, also referred to as R-, Diff-, or R/Diff locus [16]. R/Diff is located on linkage group (LG) 5 and three candidate genes have thus far been identified: rab3d [23], cdkn2ab [24], and adgre5 [25]. On that note, the three candidates for one phenotype are very distant from each other on LG5. In the presence of the R/Diff locus, the activity of Tu is restricted to a nevus-like mild local overproduction of pigment cells. Due to the location of the two genetic loci on different chromosomes, it is possible to induce malignant melanoma by separating them through chromosome segregation in defined crossing procedures [26,27]. In the classical crossing scheme, an *X. maculatus* female carrying Tu and R/Diff is crossed with a male of *X. hellerii* which does not have both loci. The resulting F1 hybrids, heterozygous for R/Diff and Tu, display an increased activity of the single copy of the Tu locus due to the half dosage of R/Diff. As a consequence, a locally limited overexpression of pigment cells occurs, which grow mildly expansive and are classified as benign pre-malignant lesions [28]. Subsequently, the F1 hybrid is backcrossed to a parental *X. hellerii*. The backcross generation separates into four genotypes: 25% Tu/wt; R/Diff/wt, which develop benign pigment cell lesions (same genotype and phenotype as the F1); 25% Tu/wt; wt/wt, developing malignant melanoma due do the full absence of R/Diff; 25% wt/wt; R/Diff/wt and 25% wt/wt; wt/wt, both with wildtype healthy phenotype because these two groups have not inherited the Tu locus with the melanoma oncogene xmrk. In previous studies, we found a high conservation of pathways involved in the development of malignant melanoma in human and *Xiphophorus* fish, showing this fish model to be a reliable tool to study genes involved in the development of malignant melanoma [29].

Studies have demonstrated the gene disrupting effect of TE transposition in Xiphophorus [30], yet analyses of total TE numbers or specific TE sequences in different Xiphophorus hybrid tissues are missing. Due to their seemingly random insertion into the genome, the TE landscape can widely vary between different organisms. Yet, the same TE families are included. To contribute to a better understanding of the role of TEs in the development and progression of melanoma, in the present work, we analyzed RNA sequencing data from 48 Xiphophorus backcross hybrids (BC2 to BC8) and compared total TE counts in healthy skin, benign lesions, and malignant tissues of hybrid fish. In our sample set, particularly TEs mapping to the region between the candidate genes, rab3d and cd97 on LG5 are disproportionately highly expressed in malignant tissue. Even though it is not possible to directly transfer the results of individual TEs to the human genome, a general upregulation of TE expression could be observed and can be used to further study the underlying regulatory mechanisms.

## 2. Materials and Methods

### 2.1. Fish

All fish were raised in the aquarium of the Biocenter of the University of Würzburg and kept under standard conditions [30]. The crossing experiments and sampling were performed in accordance with the guidelines of the German animal welfare law (Tierschutzgesetz §11, Abs. 1, Nr. 1) and approved by the government of Lower Franconia (permit number 568/300-1870/13).

The following materials were used:

1. Malignant melanoma 407: Tumors collected from backcross hybrids of *X. maculatus* and *X. hellerii* (pedigree ID WLC 407). Repeated backcrosses (>100) of Tu-allele (mdlSd-xmrkB) bearing hybrids with *X. hellerii* resulted in an isogenic line that has a copy of the mdlSd-xmrkB region from the X chromosome of *X. maculatus* in the genetic background of *X. hellerii*. Spontaneous development of malignant melanoma occurs in 50% of each backcross generation.
2. Malignant melanoma 1844: Tumors of the WLC 1844 strain are phenotypically similar to those of WLC 407 fishes. The WLC1844 strain is genetically analogous to the WLC 407 strain, except that xmrk is expressed from a spontaneously translocated fragment of the mdlSd-xmrkB region from the X chromosome of *X. maculatus* to an autosome of *X. hellerii*. These fish develop malignant melanoma.
3. Benign lesions: Pre-malignant benign pigmented lesions were taken from individuals of the second to eighth generations of backcross (BC) hybrids produced in an independent series of successive crosses from F1 hybrids of a male *X. maculatus* JP 163A with a female *X. hellerii*. F1 hybrid females with Tu-allele (mdlSd-xmrkB) were backcrossed to *X. hellerii* males for several generation (WLC 1337) and segregant fish with benign lesions having the LG5 located R/Diff locus were used for further backcrosses.
4. Control tissue: Healthy skin, fin, eye, brain, liver, and gills were collected from the 407 mutant strain (WLC 6608). This strain is derived from the WLC 407 strain described above when a spontaneous mutation occurred that disrupted expression of the mdlSd-xmrkB locus. This mutant fish did not develop any pigment lesions and was further backcrossed to *X. hellerii*. All fish of this line present a healthy phenotype.

### 2.2. Sequencing

TE expression analysis was based on a meta-analysis of sequencing data from 48 individual Xiphophorus hybrids. The data resulted from four independent custom sequencing runs performed with the Illumina TruSeq library preparation system (Illumina, Inc., San Diego, CA, USA) at BGI (Shenzhen, China) between the years 2013 and 2023. Depending on the run, 20–60 million paired-end reads were sequenced with a length ranging from 90 to 150 bp.

### 2.3. Expression Profile

Raw reads were filtered for contamination and low-quality reads (Q20) and adaptor sequences were removed by BGI. Clean reads were aligned to a fish-specific transposon database which contains 4090 consensus sequences of TEs found in 19 different fish species [31] using Bowtie2 (default settings) [32]. TEs present at multiple locations in the genome were collapsed and only one representative sequence was given as a consensus sequence. Of these consensus sequences, 818 were first identified in *X. maculatus*. The resulting dataset included 18 malignant melanoma and 20 benign (premalignant) lesions tissue samples from 38 individual Xiphophorus fish. One dataset additionally included adjacent healthy skin from 10 fish with benign phenotype (Tu/-; R/Diff). Resulting read counts were calculated and differential expression of TEs was detected using the R/Bioconductor package DESeq2 [33]. TEs with a Basemean of >10 were considered as expressed. Expressed TEs with a log2Fc > 1 and a significant *p*-value (significance level: 0.05) were defined as differently expressed TEs. Significance levels in plots are shown as “*” for *p* < 0.05, “**” for *p* < 0.01 and “***” for *p* < 0.001. Basemean was calculated as (mean (normalized read counts (groupA)) + mean (normalized read counts (groupA)))/2.

In order to more precisely analyze TE expression on LG5, reads intersecting with repetitive regions or TEs resulting from RepeatMasker (https://www.RepeatMasker.org/, (version open-4.0.7) using our transposon database as custom library) for *X. maculatus* and *X. hellerii*, respectively, were calculated and compared. Functional protein association networks were found using STRING [34]. Genomic regions were visualized by the Integrative Genomics Viewer (IGV, https://igv.org/, accessed on 27 March 2024, Version 2.11.3 11/03/2021).

### 2.4. RNA-Seq Validation and qPCR

To test the reliability of the expression analysis, genomic locations of four selected consensus TE sequences were detected using the program TE-Aid (https://github.com/clemgoub/TE-Aid/blob/master/README.md/, accessed on 27 March 2024, v.0-dev). The longest corresponding sequence in the genome was used to confirm expression by qPCR. For expression analysis, brain, gill, eye, liver, healthy skin, and fin from benign and pre-malignant lesions, and malignant melanoma were harvested from adult Xiphophorus hybrids. RNA was extracted using TRIZOL reagent (ThermoFisher Scientific, Waltham, MA, USA) and processed as described previously [35]. Spectrophotometry (NanoDrop ND-1000, PeQLab Biotechnology GmbH, Erlangen, Germany) was used to measure purity and concentration. Reverse transcription was performed using the RevertAid First Strand cDNA Synthesis kit (ThermoFisher Scientific). In total, 25 ng of cDNA was added to a 25 μL reaction tube containing a SYBR-green (Sigma Aldrich Chemie GmbH, St. Louis, MO, USA)-enriched master mix. For PCR primers, see Supplementary Table S1. An Eppendorf Mastercycler EP Gradient S was used with the following settings: 2 min at 95 °C followed by 40 cycles of 95 °C for 15 s, 60 °C for 30 s, and 72 °C for 30 s. After completion of the reaction, the expression of each sequence was normalized to the expression levels of elongation factor 1 (efa1). Relative expression (deltaCT) was determined as described [36]. Significance of expression differences of more than two compared tissue types were calculated using the Kruskal–Wallis rank sum test (post hoc test: Tukey and Kramer (Nemenyi)) from the R package PMCMR. To test the limits of the detection of differential expression by RNA-seq, we selected TEs that had only low fold change values (log2FC −0.59 to 0.60) and low expression (~20 reads) to high expression (270 to 544 reads) for qPCR.

## 3. Results and Discussion

In this study, we explored transposon expression during development of malignant melanoma compared to benign pre-malignant lesions on a genome-wide level in *X. maculatus*/*X. hellerii* hybrids. For this, we analyzed transcriptomic reads of 20 benign and 18 malignant lesions in hybrids as well as 10 healthy skins as control.

### 3.1. Overall Expression Profile

**Figure 1:**
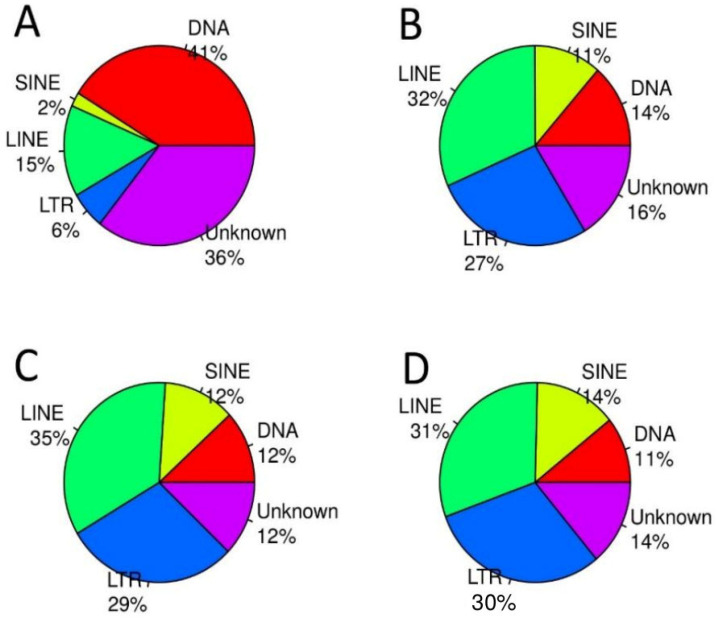
Pie chart of percentage of fish TE families included in the fish-specific consensus reference library (**A**) and percentage of families of expressed TEs found in RNA-seq data of healthy control tissue (**B**), benign lesions (**C**), and malignant lesions (**D**) of adult Xiphophorus hybrids.

As reported in several cancer types, malignant transformation resulted in increased transcription of TEs [37]. To get information about global expression of TE families of benign and malignant lesions, we mapped the RNA-seq reads of all samples to a reference database with 4090 fish TE consensus sequences. Subsequently, read counts were calculated. Within this reference, consensus sequences of DNA transposons represented the largest group (41%), followed by 15% LINE, 6% LTR, and 2% SINE TEs (Figure 1A). Unclassified TEs accounted for 36%. To compare total TE expression in the different entities, normalized read counts were calculated in RNA-seq data of healthy skin (control), benign lesions, and malignant melanoma. In our sample, transcriptomes 2229 TE consensus sequences were considered to be expressed. Only if a TE consensus sequence was expressed in at least two samples, was it considered for further analysis. In total, 255 expressed sequences showed consistent differential expression between benign and malignant tissue. Of these, 209 were upregulated in malignant tissue, whereas only 46 showed downregulation. In contrast to the reference TE family abundances in the genome, our transcriptomic data exhibited a significant increase in the expression of class I transposons indicating activity in all three tissue types (Figure 1), whereas DNA TEs accounted for less than 14%.

**Figure 2:**
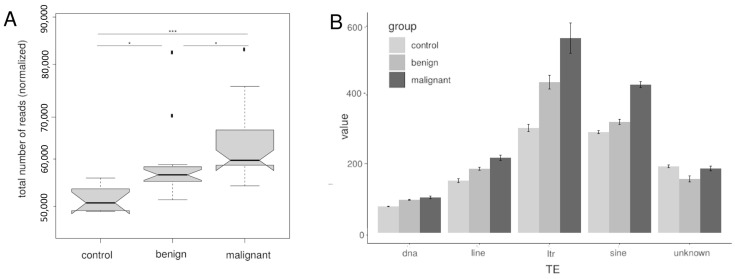
(**A**) Total number of normalized read counts calculated in RNA-seq data of healthy skin (control), benign, and malignant lesions (control: n = 10, benign: n = 22, malignant: n = 26). (**B**) Bar-plot of median normalized read counts of TEs expressed in control, benign, and malignant tissue samples of different TE families. Nemenyi-tests for multiple comparisons: *** *p* < 0.001, * *p* < 0.05.

The highest number of TE read counts was recorded in malignant melanoma followed by benign lesions and healthy skin (Figure 2A).

A significant difference was found between all three groups. These results confirm an increasing overall TE expression during tumor development. All characterized TE families showed increased expression with progression to malignancy with larger differences in class I transposons, but downregulation in benign samples in uncharacterized TEs. This is in line with the observation in other species. However, in human cancers, LINE elements are more predominant, where increased expression of LINE elements in particular was found to be responsible for genetic damage and thus genomic instability [38,39]. In humans, LINE expression became higher with increased malignancy. In the presently studied fish samples, LTR and SINE TEs were more abundant than LINE TEs. This may be simply related to the different overall TE composition in the genomes of fish and humans. In fish, more different TE types are present in the genome but with lower abundancy, while in humans TEs show lower variability but higher abundancy [31].

To evaluate the transcriptomic data, regulation as well as expression levels of four TEs (Table S1) were selected for more in-depth analyses by qPCR. We chose TEs which showed moderate expression and a low fold change to better assess limitations of RNA-seq. Expression levels were determined in malignant melanoma, benign lesions, and healthy control tissue from Xiphophorus hybrids. In general, the qPCR results confirmed the RNA-seq data on regulation of all four transposable elements (see Supplementary Data), indicating a good reliability of the RNA-Seq data.

### 3.2. Comparison of Benign Melanocytic Lesions and Adjacent Healthy Skin in the Same Individual

**Figure 3:**
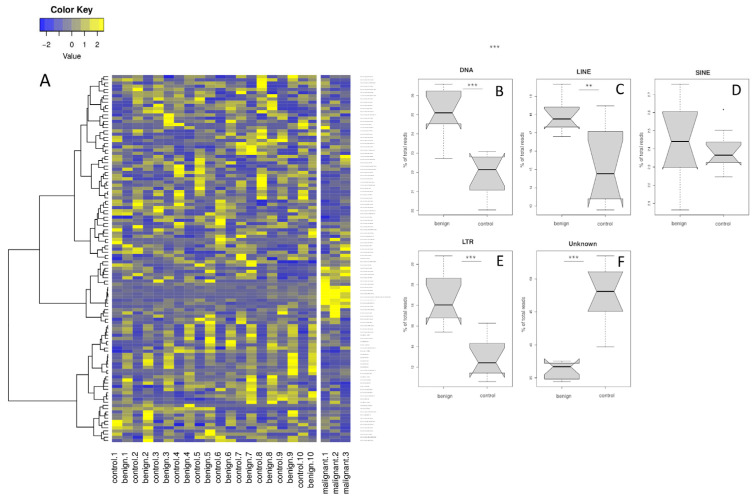
(**A**) Heatmap of differentially expressed TEs in control and adjacent benign lesions of 10 fish and malignant samples (n = 3) from the same sequencing run. Equal numbers for control and benign samples indicate identical individuals. Transcripts with higher expression levels are shown in yellow, while lower expression is shown in blue. Right panels: Boxplots of differential expression of control (skin) and benign lesions according to transposon family type: DNA (**B**), LINE (**C**), SINE (**D**), LTR (**E**) transposons, and uncharacterized TEs (**F**). Student’s *t*-test: * *p* < 0.05, ** *p*< 0.01, *** *p* < 0.001.

To assess individual differences, we tested a sample set including benign pigment lesions and an adjacent healthy control skin from 10 individuals and three malignant melanoma samples from the same backcross generation (Figure 3A).

TEs were upregulated in benign as compared to control samples. TEs high in malignant melanoma display a reproducible pattern between groups, but tend to have low read counts and small fold changes. In contrast, TEs downregulated in benign lesions vary between samples and tend to have high read counts and relatively strong down-regulation.

### 3.3. Transposable Elements on LG5 and LG21

In previous studies, R/Diff, the putative autosomal regulator locus for xmrk, has been located on LG5 [24]. With three different and unrelated candidate genes located over a large distance on this chromosome, the molecular identity and mode of action of R/Diff is unclear. This motivates approaches which consider that R/Diff action is not monogenic but polyfactorial from a larger segment on this chromosome.

According to the crossing scheme, fish with either Tu/-; R/Diff/- (benign lesions) or Tu/-; -/- (malignant melanoma) were selected. Thus, all fish have the xmrk gene as the critical component of the Tu locus. In contrast, only fish with benign lesions are heterozygous for LG5 regions derived from *X. maculatus* [40]. In our dataset, we could confirm that in benign samples regions of LG5 containing all three candidate genes for R/Diff were derived from *X. maculatus*. In our inbred lines this region, heterozygous for *X. maculatus* and *X. hellerii*, approximately covered 25 Mb from the 5′ end of LG5 [41] all three candidate genes.

**Figure 4:**
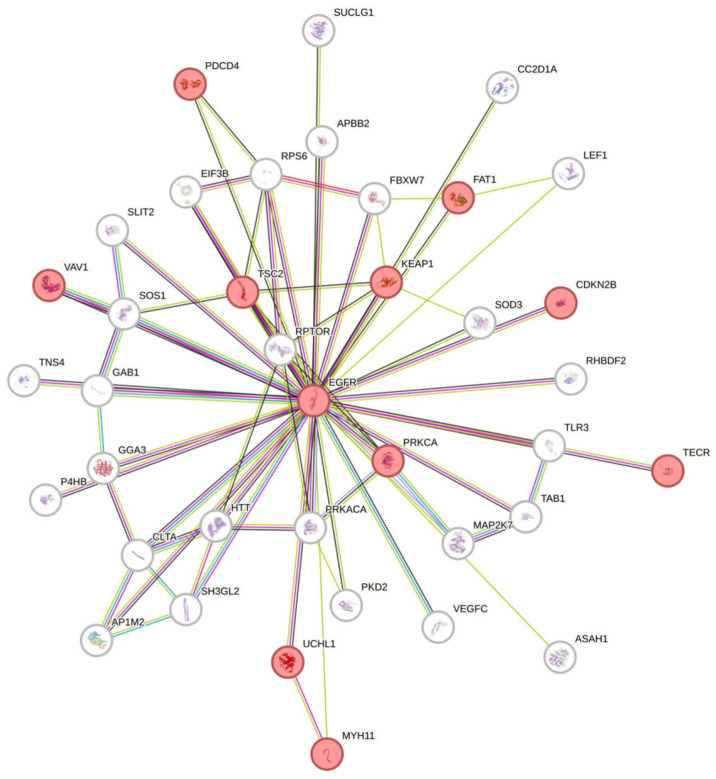
Functional protein association networks using STRING. Genes on Xiphophorus LG5 with known interaction with EGFR. Edges represent protein–protein associations. Associations taken into account are given under Getting started—STRING Help (string-db.org). Thicker edges represent higher confidence. Genes marked in red have known associations to cancer development.

On LG5, there are a large number of genes related to DNA repair, DNA replication, cell cycle regulation, or genes important for mRNA splicing. In Figure 4, genes with known association to EGFR are shown. Incompatibilities or differential epigenetic regulation resulting from hybridization, which affect LG5 in particular, may have severe consequences for gene expression and thus for the development of either benign lesions or malignant melanoma.

To examine transposons on the putative regulatory locus on LG5 more closely, read counts with overlap to TEs and repetitive sequences derived from RepeatMasker for *X. maculatus* and *X. hellerii* genomes were calculated for all samples and compared between parental species. Using RepeatMasker against both reference genomes resulted in a higher number of masked bases in *X. maculatus* than in *X. hellerii*. In a previous study, it has been shown that TE annotations of *X. maculatus* and *X. hellerii* showed similar TE coverage and equal distribution between class I and class II elements [41]. This difference between our result and the previous study might be due to different Repeatmasker settings and the use of our transposon reference database as a custom library.

In accordance with the results of the alignment to the consensus sequences there is a higher total number of reads mapping to TE regions in malignant samples than in benign samples, however, with an extreme increase in the region between candidate genes on LG5 (Table 1, ratio (mal/ben). An explanation for the lower number in benign lesions might be some trans-suppressing effect by *X. maculatus*-derived sequences. In Drosophila, it has been suggested that TE derepression in interspecific hybrids might be caused by an incompatibility of piRNA pathway genes rather than species-specific differences in TE-derived piRNAs [42]. Proteins involved in small RNA biogenesis located in hybrid regions are Phospholipase D Family Member 6 (pld6) on LG5, next to one of the candidate genes, rab3d and Drosha Ribonuclease III on LG21. *X. maculatus* has a gene duplication of drosha, next to xmrk. Due to the selection for fish bearing xmrk, most likely hybrids have at least one copy originating from *X. maculatus*. In Drosophila an impaired piRNA-mediated TE transcriptional repression has been shown [43].

Comparing the proportion of reads mapping to repeatmasked regions in *X. hellerii* and *X. maculatus*, most LGs have a higher number of reads mapping to the *X. hellerii* genome. Even though in higher backcross generations, most sequences are derived only from *X. hellerii*, reads mapping to *X. maculatus* can be explained by a high similarity of expressed Tes in both parental species and only a smaller proportion of unique sequences in *X. hellerii*. In contrast, on LG5 (benign samples) and LG21 (all samples), Tes can be derived from both species. Therefore, unique Tes from either parent can be expressed. However, on LG21 with the xmrk region from *X. maculatus* and even more in the region between candidate genes rab3d and CD97 antigen-like on LG5, there are ~2-fold more reads with higher similarity to *X. maculatus* (Table 1). One explanation for this bias towards *X. maculatus* reads is that transcriptional silencing of active TEs is attained by DNA methylation. Unlike in mammals, where there is a genome-wide DNA methylation reprogramming during embryogenesis [44], the parental species methylation patterns have been shown to be stable in several fish species, e.g., in medaka or Xiphophorus [45,46,47]. Consequently, the *X. maculatus*-specific methylation patterns on LG5 and LG21 would be carried on in the backcross hybrids. This inference has to be substantiated by methylome sequencing in future studies. Furthermore, it has been shown that gene and TE expression in somatic cells can be affected by DNA methylation through piRNAs [48]. In a previous study, we compared the piRNA cluster expression of malignant versus benign control tissue. This revealed that 10-fold more clusters were down-regulated (fold change > 4-fold) than up-regulated in the malignant tissue samples [49]. Based on piRNA read counts, it was even possible to distinguish between benign and malignant tissue samples.

**Figure 5:**
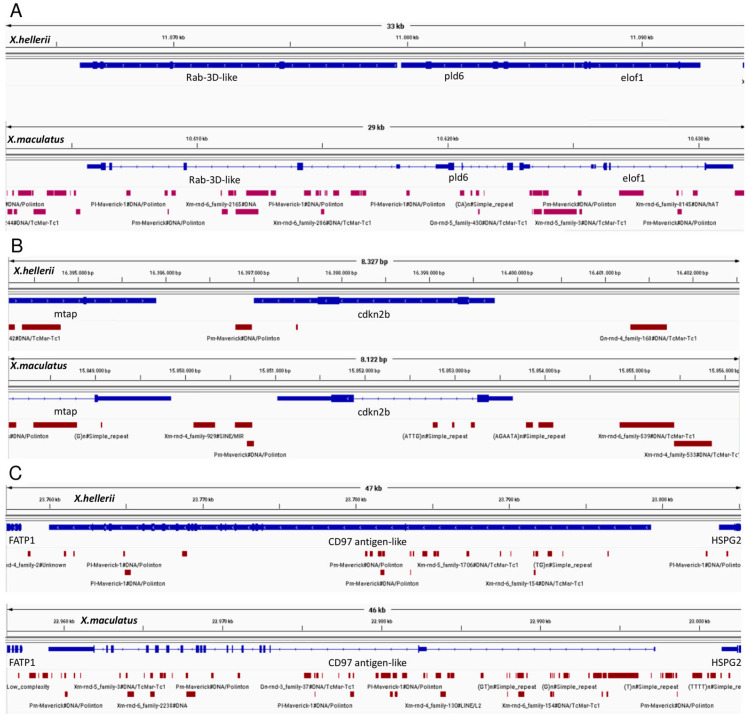
Integrative Genomics Viewer (IGV): region around the three candidate genes rab3d-like (**A**), cdkn2a/b (**B**), and CD97 antigen-like (**C**). In the upper panel RepeatMasker-annotated TEs in *X. hellerii* are shown as red bars. In the lower panel panel, RepeatMasker-annotated TEs in *X. maculatus* are shown.

In Figure 5, TEs near the R/Diff candidate genes are shown. In all three regions, more TEs were found by RepeatMasker in *X. maculatus* than in *X. hellerii*. In the rab3d genomic region, TEs are completely missing in *X. hellerii*. Even though almost all TEs are located outside coding sequences, especially in *X. maculatus*, there are TEs in intronic or promotor regions, which can affect gene expression either by altered transcription factor binding sites or methylation sites [48,50]. Differential expression between malignant melanoma vs. benign lesions has been shown for cdkn2ab [51] and rab3d [23] and for adgre 5 between invasive melanoma and nevus-like spots [25]. This hypothesis is strengthened by the fact that melanocytes undergo progressive hypomethylation during carcinogenesis and that similar processes were described in benign lesions [52,53].

## 4. Conclusions

In summary, we constructed a specific TE expression profile from which a variety of TEs emerged that were differentially expressed in benign and malignant Xiphophorus tissue. It was shown that the parental genome origin for the expression of the TEs differed in the hybrids. Furthermore, we identified an increased TE activity in malignant Xiphophorus tissue compared to benign lesions and healthy skin, which indicates a correlation between the epigenetic dysregulation rate and the TE expression as well as a basal TE transcription rate in healthy skin. These results are in agreement with a study that reported increased transposon expression in several human tumor types [54], however, melanoma was not studied. We were able to experimentally verify the increase in expression of four TEs in malignant tissue, potentially contributing to genetic instability.

The maintenance of genome stability is among the most important transcriptional inhibition mechanisms of TEs under physiological conditions. Consequently, suspension of these processes in pathological process may imply reactivation of many TEs, which may directly affect their expression. Our results emphasize a general impact of TEs in the development of malignant melanoma which can also be observed in humans. However, a direct transfer of the results is difficult, and many questions remain to be investigated in further studies, such as the underlying mechanisms regarding the identification of epigenetic factors, tumor-specific insertions, and their potential influence on neighboring genes.

## Figures and Tables

**Table 1:** Reads overlapping to repeatmasked regions in *X. maculatus* or *X. hellerii*. Numbers represent the mean of mean (overlapping reads per TE). Only TEs with the sum (all reads per TE) > 10 were included.

	xma	xhe	Ratio (xhe/xma)	Benign Total	Malignant Total	Ratio (mal/ben)
LG5 total	142.13	214.06	1.51	133.77	203.70	1.52
LG5 region between candidate genes	52.90	22.83	0.43	12.57	68.58	5.46
LG21	113.38	58.58	0.52	77.47	95.63	1.23
LG1-4,6-20 (±SD)	153.87 (±55.94)	204.34 (±109.04)	1.33 (±0.47)	148.70 (±127.55)	196.91 (±72.91)	1.41 (±0.26)

## Data Availability

All samples are deposited at the NCBI sequence read archive (SRA) under the ID: PRJNA1091453.

## Supplementary Materials

The following supporting information can be downloaded at: https://www.mdpi.com/article/10.3390/genes15050620/s1, Table S1: Sequences of qRT-PCR primers, Figure S1: Relative expression (ΔCT) of 4 TEs in different Xiphophorus tissues. (A1, A2) Relative expression of Xma_rnd-5_family-531_DNA/hAT-Charlie (healthy skin: n = 10, benign lesions: n = 4, 1844 malignant: n = 10, 407 malignant: n = 7). (B1, B2) Relative expression of Gaf_rnd-5_family-1020_Unknown (healthy skin: n = 9, benign lesions: n = 5, 1844 malignant: n = 9, 407 malignant: n = 8). (C1, C2) Relative expression of Oni_rnd-6_family-283_DNA/hAT (healthy skin: n = 4, benign lesions: n = 4, 1844 malignant: n = 7, 407 malignant n = 8). (D1, D2) Relative expression of Cse_Piler_54.65_DNA/hAT-Charlie (healthy skin: n = 5, benign lesions: n = 3, 1844 malignant: n = 3, 407 malignant: n = 4).
