# Supplementary material for: Transposable Element Expression Profiles in Premalignant Pigment Cell Lesions and Melanoma of Xiphophorus
Source: Genes (Basel). 2024 May 14;15(5):620. doi: 10.3390/genes15050620 (PMC11121471; doi:10.3390/genes15050620)
Supplement: Supplementary file 1 [file genes-15-00620-s001.zip › genes-2962856-supplementary.pdf]

Supplement

Efa1_for	AGTGAAATCCGTGGAGATGC
Efa1_rev	ATCTGACCTGGGTGGTTCAG
Xma_rnd-5_family-531_DNA/hAT-Charlie_for	TCAAGTACCCCCTGACACGGGC
Xma_rnd-5_family-531_DNA/hAT-Charlie_rev	CTTCCTGGTGGTCACCGTCTTCTG
Gaf_rnd-5_family-1020_Unknown_for	ACATGCTGAGAGTGAAAATCCT
Gaf_rnd-5_family-1020_Unknown_rev	CAAACAGGAAGGGCGGGAC
Oni_rnd-6_family-283_DNA/hAT_for	ACTGGGAGGTTGAGTGGAGA
Oni_rnd-6_family-283_DNA/hAT_rev	TGTGCCAGACAGAGTAACCA
Cse_Piler_54.65_DNA/hAT-Charlie_for	TCAGTTTGAGTTGGCCGAGT
Cse_Piler_54.65_DNA/hAT-Charlie_rev	GGGAAAGGTTTTGCTCGCAG

Table S1: Sequences of qRT-PCR primers

Expression profiling of selected TEs in melanocytic lesions

Expression levels were in good correlation to sequencing results and qPCR with Oni_rnd-6_family-283_DNA/hAT being highly expressed and Cse_Piler_54.65_DNA/hAT-Charlie having low expression levels (Supplementary figure 1, A-D). Compared to benign tissue, Xma_rnd-5_family-531_DNA/hAT-Charlie showed a significant increase in expression in malignant melanoma of the 407 strain (p-value: < 0.001), while decreased expression was observed in melanoma tissue of the 1844 strain. Gaf_rnd-5_family-1020_Unknown showed increased expression levels in all types of melanocytic tissues with a strong overexpression in the 407 melanoma. In case of Oni_rnd-6_family-283_DNA/hAT and Cse_Piler_54.65_DNA/hAT-Charlie a decreased expression was detected in malignant melanomas of both strains compared to benign lesions. To gain a better understanding of the expression patterns, qPCR was extended to additional non-tumorous *Xiphophorus* tissue types including brain, eye, gills, and liver. The lowest expression of Xma_rnd-5_family-531_DNA/hAT-Charlie was determined in liver tissue. Slightly increased values were found in gills and eye, whereas the highest

expression was measured in brain. Comparison of expression in malignant melanoma, benign lesions and healthy skin showed an increase in expression in the 407 malignant tissue (figure 8A). A similar expression pattern between tissue types was seen for Gaf_rnd-5_family-1020_Unknown (Figure 8B). With the exception of the 407 tumor strain the lowest expression was recorded in liver tissue. Slightly increased expression could be detected in gills, while the highest expression was measured in brain tissue. The lowest expression of Oni_rnd-6_family-283_DNA/hAT was recorded in liver tissue. In contrast, a slightly increased expression was determined in brain and eye of all investigated fish lines. The highest expression levels were seen in gill tissue. Cse_Piler_54.65_DNA/hAT-Charlie showed an overall low expression. With the exception of brain tissue, ΔCT values > 7 were observed within the different entities.

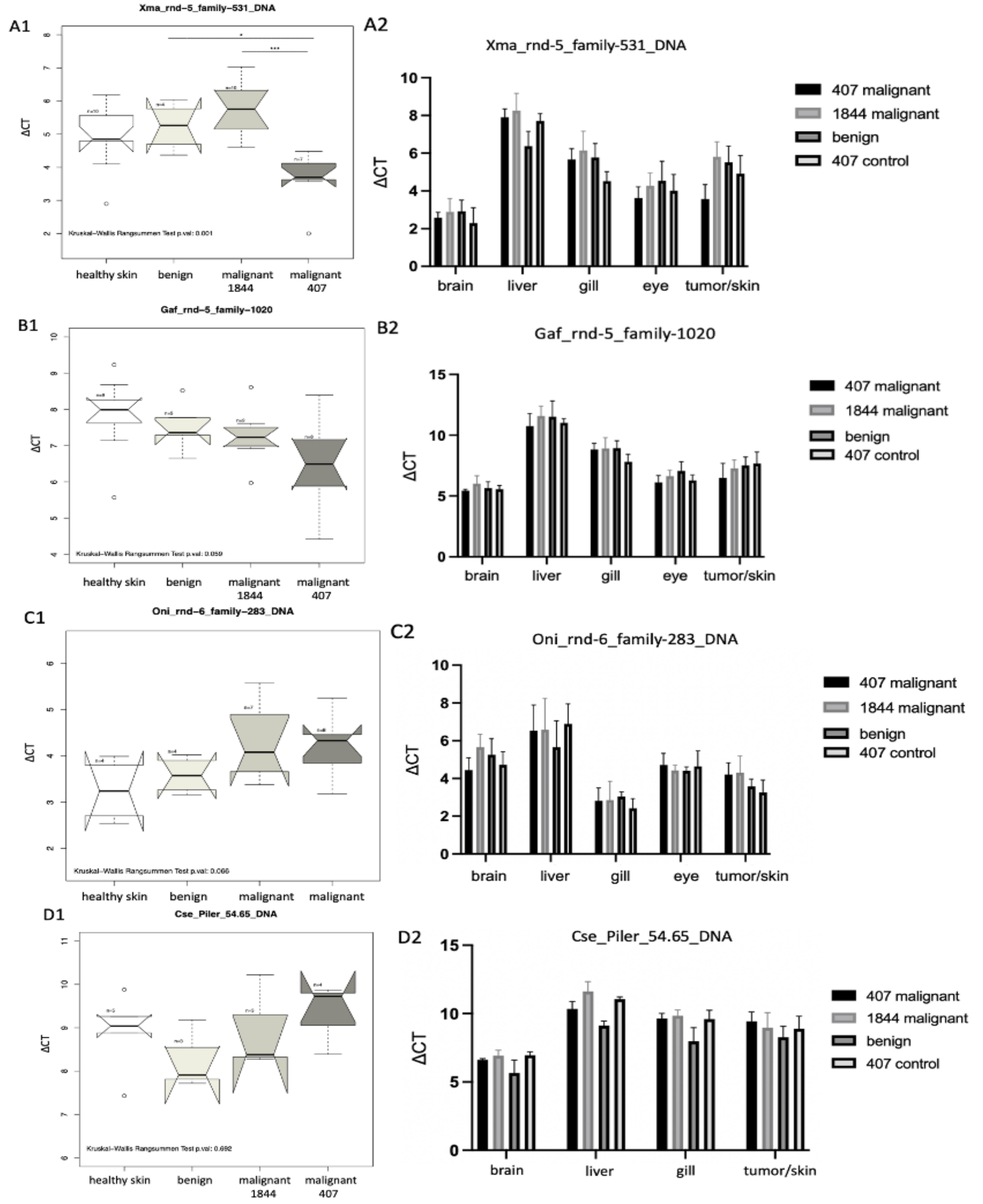

Figure S1: Relative expression (ΔCT) of 4 TEs in different Xiphophorus tissues. *(A1, A2)* Relative expression of Xma_rnd-5_family-531_DNA/hAT-Charlie (healthy skin: n=10, benign lesions: n=4, 1844 malignant: n=10, 407 malignant: n=7). (B*1,* B2) Relative expression of Gaf_rnd-5_family-1020_Unknown (healthy skin: n=9, benign lesions: n=5, 1844 malignant: n=9, 407 malignant: n=8). *(C1, C2)* Relative expression of Oni_rnd-6_family-283_DNA/hAT (healthy skin: n=*4,* benign lesions: n=4, 1844 malignant: n=*7,* 407 malignant n=*8*). (D1, D2)

Relative expression of Cse_Piler_54.65_DNA/hAT-Charlie (healthy skin: n=*5*, benign lesions: n=*3,* 1844 malignant: n=*3,* 407 malignant: n=*4*).
